# Electrocortical Responses to Emotional Stimuli in Psychotic Disorders: Comparing Schizophrenia Spectrum Disorders and Affective Psychosis

**DOI:** 10.3389/fpsyt.2018.00586

**Published:** 2018-11-16

**Authors:** Adam J. Culbreth, Dan Foti, Deanna M. Barch, Greg Hajcak, Roman Kotov

**Affiliations:** ^1^Department of Psychological and Brain Sciences, Washington University in Saint Louis, Saint Louis, MO, United States; ^2^Department of Psychological Sciences, Purdue University, West Lafayette, IN, United States; ^3^Departments of Psychiatry and Radiology, Washington University in Saint Louis, Saint Louis, MO, United States; ^4^Department of Psychology, Florida State University, Tallahassee, FL, United States; ^5^Department of Psychiatry, Stony Brook University, Stony Brook, NY, United States

**Keywords:** psychosis, electrophysiology, affective functioning, mood disorder, schizophrenia, transdiagnostic

## Abstract

Emotion dysfunction has long been considered a cardinal feature across psychotic disorders, including schizophrenia and affective psychosis. However, few studies have used objective markers of emotional function to compare psychotic disorders to one another, and fewer studies have examined such markers within a longitudinal framework. Here, we examine one objective marker of emotional responsivity, the late positive potential (LPP), which is a centro-parietal event-related potential (ERP) that tracks the dynamic allocation of attention to emotional vs. neutral stimuli. We used the LPP to characterize abnormal emotional responsivity by relating it to negative, depressive, and psychotic symptoms among two clinical groups: individuals diagnosed with affective psychosis and individuals with schizophrenia. We also used a long-term longitudinal framework, examining concurrent associations between LPP amplitude and symptom severity, as well as prospective associations with symptoms 4 years later. Participants were 74 individuals with psychotic illness: 37 with schizophrenia spectrum disorders and 37 with a primary affective disorder (psychotic bipolar disorder, psychotic depression). There were no mean-level differences in LPP amplitude between the schizophrenia spectrum and primary affective psychosis group. In the primary affective psychosis group, reduced LPP amplitude was associated with greater depressive, negative, and psychotic symptom severity, both concurrently and at follow-up; associations between LPP and symptoms were not observed within the schizophrenia spectrum group. This pattern of results suggests that the neural correlates of emotion dysfunction may differ across psychotic disorders. One possibility is that schizophrenia is characterized by a decoupling of symptom severity and emotional processing. Such findings underscore the importance of analyzing transdiagnostic samples to determine common or specific symptom relationships across various patient populations.

## Introduction

Emotion dysfunction is present in various forms across psychotic disorders, manifesting as negative (e.g., flattened affect), and depressive symptoms (e.g., anhedonia), and even potentially contributing to psychotic symptoms (e.g., paranoia). However, it is currently unclear whether abnormal emotional processes differ across psychotic disorders, and whether emotional processes show differential relationships to symptoms, potentially yielding clinically distinct phenotypes. For example, it is unclear whether emotional processes show differential relationships to symptoms for individuals diagnosed with a primary affective disorder (e.g., major depressive disorder or bipolar disorder) with psychosis compared to those diagnosed with a schizophrenia spectrum disorders. In order to answer such questions, one recent push in clinical science has been the identification of objective neural markers which demonstrate relationships with symptoms (1, 2). Such markers have the potential to objectively index symptom severity, assist in differential diagnosis, and aid in further understanding the mechanisms that underlie emotional disturbances across psychotic disorders (1–3).

One potential neural marker for emotional dysfunction is the Late Positive Potential (LPP). The LPP is an event-related potential (ERP) component typically evident ~300 ms after stimulus onset and at centro-parietal sites. LPP amplitude is increased following pleasant and unpleasant compared to emotionally neutral stimuli, including pictures, words, and faces (4–9). The LPP is thought to index sustained attention to emotionally-salient stimuli (10, 11), and thus may be particularly relevant to symptoms of psychiatric disorders that relate to emotion dysfunction (9). Indeed, several reports examining the LPP in psychiatric populations have suggested that it is associated with depressive symptoms (12–15). For example, LPP amplitude to threatening and rewarding pictures was shown to be reduced in depressed individuals compared to controls (15). Foti and colleagues reported similar findings of lower LPP amplitude to threatening faces in depressed individuals compared to controls (12). Further, some evidence suggests those at-risk for developing depression demonstrate reduced LPP amplitude to pleasant, unpleasant, and neutral stimuli when compared to lower-risk samples (14, 16). In regards to schizophrenia, LPP amplitude to pleasant, but not unpleasant pictures has been found to be reduced in those with schizophrenia vs. controls (13). However, there is also evidence of normative LPP amplitude to affective pictures among those with schizophrenia (17). Thus, consistent evidence suggests lower LPP compared to controls in those with primary affective disorders while evidence for lower LPP in schizophrenia spectrum disorders is less consistent.

While previous reports have been informative in establishing LPP-to-symptom associations within particular diagnostic categories, few studies have examined the LPP across psychotic disorders. In one exception, Horan and colleagues measured LPP during a motivational gradient task where cues signaling potential monetary gain or loss appeared to loom progressively closer to the observer (18). They found differences in temporal dynamics of the LLP between individuals with bipolar disorder and those with schizophrenia. Specifically, the bipolar group was characterized by an early escalation of LPP during cue presentation, whereas the schizophrenia group showed diminished escalation of LPP to either cue (18). While this study was informative in further understanding differences in the temporal dynamics of motivational experience across psychotic disorders, additional samples including a wider variety of psychotic disorders could be useful in better understanding how emotional processes might differ across affective psychosis and schizophrenia spectrum disorders. In addition, the majority of studies assessing LPP in psychiatric populations have been cross-sectional. Such studies are limited in their ability to determine whether abnormalities in LPP are shaped by current symptoms, or if such deficits are more enduring or trait-like. Thus, longitudinal designs are needed to better characterize the nature of LPP deficits and how they may differ across various psychotic disorders (19).

In the current study, we examined relationships between LPP and symptoms of emotional dysfunction within a clinically heterogeneous sample of individuals with psychotic illness. To test the potential transdiagnostic nature of LPP-to-symptom relationships, we contrasted two groups: individuals with schizophrenia spectrum disorders and individuals with a primary affective disorder with psychosis (i.e., psychotic bipolar disorder, psychotic depression). We also utilized a longitudinal approach, examining the relationships between LPP amplitude and concurrent symptom severity as well as symptom severity at a 4-year follow-up. Using these data, we tested two competing hypotheses: (1) Across psychotic disorders, the LPP would demonstrate both cross-sectional and lagged associations of similar magnitude with depressive, negative, and psychotic symptoms, consistent with transdiagnostic, temporally stable (i.e., trait-like) association; (2) LPP amplitude may differ between schizophrenia and affective psychosis (i.e., lower LPP amplitude in affective psychosis) and LPP-to-symptom relationships exhibit diagnostic specificity with more robust associations in those with a primary affective disorder with psychosis than with a schizophrenia spectrum disorder. This second hypothesis is consistent with strong evidence of diminished LPP in those with major depressive disorder (12, 15), but inconsistent with previous work in schizophrenia (20).

## Methods

### Participants

Participants were recruited from the Suffolk County Mental Health Project (21, 22), an epidemiologic longitudinal study of first admission psychosis. The sampling frame consisted of consecutive first admissions with psychosis to the 12 psychiatric inpatient facilities in Suffolk County, N.Y., from 1989 to 1995. We specifically included individuals within the sample that met diagnostic criteria for schizophrenia, schizoaffective disorder, bipolar disorder with psychosis, or major depressive disorder with psychosis, at a study session 15 years after first admission. The current manuscript is one of multiple published with data collected from this cohort (23–27). Inclusion criteria were: (1) presence of psychosis; (2) IQ > 70; (3) age between 15 and 60 years at admission; (4) resident of Suffolk County; (5) ability to provide informed consent. This study was approved by the Institutional Review Board at Stony Brook University. EEG was collected ~15 years after first admission from 74 participants: 37 participants with 10-year follow-up consensus diagnoses of schizophrenia spectrum disorders (SZ: 23 schizophrenia, 14 schizoaffective disorder) and 37 participants with affective psychosis (AP: 27 bipolar disorder, 10 major depressive disorder). Across our sample of 74 individuals, we had ~95% power to detect an effect size of *f* ^2^ = 0.15 in a model with three independent variables. Similar effect sizes were found between depression symptom severity and LPP to threatening compared to neutral faces across a sample of individuals with major depressive disorder and healthy controls (12).

Although schizoaffective disorder is characterized by prominent affective disturbances, we included these individuals within the schizophrenia spectrum disorders group instead of the primary affective psychosis group. Previous work has suggested that schizoaffective disorder is better characterized as part of the schizophrenia spectrum than of primary mood pathology (28).

### Clinical assessment

Clinical assessments were conducted concurrently with EEG acquisition (i.e., 15 years after first admission) and at 19-year follow-up. At each assessment, participants were evaluated by master's level interviewers using the Structured Clinical Interview for DSM-IV-TR Axis I Disorders (SCID-IV) (29), the Scale for the Assessment of Positive Symptoms (SAPS) (30), and the Scale for the Assessment of Negative Symptoms (SANS) (31). For the SAPS and SANS, four subscales were identified: reality distortion and disorganization, avolition, and inexpressivity (32). Current depressive symptom severity was operationalized by summing scores for the nine criterion A items for current major depressive disorder on the SCID-IV administered without skip-outs. Items were scored on a 1–3 scale, and the SCID Depression score range from 9 to 27 (33). Finally, a measure of general cognitive ability, reading subtest of the Wide Range Achievement Test-3rd Edition (WRAT-3) (34), was collected at the 19-year time point.

### Stimulus materials and task

Participants passively viewed 130 images selected from the NimStim set (35)^1^. Twenty-six images were presented for each of five facial expressions: happy, sad, angry, afraid, and neutral. These images depicted 26 different actors (13 male, 13 female), such that each actor appeared for each of the five emotional face categories^2^. Each face was presented once, in a pseudorandom order for each participant. Images were presented for 1,000 ms, followed by an inter-trial interval that varied randomly from 1,000 to 1,500 ms. Participants were instructed to simply view the images.

### EEG recording, processing, and data reduction

EEG was recorded using the Active Two BioSemi System, sampled at 1,024 Hz using 34 scalp electrodes and two mastoid electrodes. The electro-oculogram was recorded from four facial electrodes. Offline analysis was performed using Brain Vision Analyzer software (Brain Products). Data were re-referenced to the mastoid average and band-pass filtered from 0.1 to 30 Hz. The EEG was segmented on each trial in a window of −200 to 1,000 ms surrounding image onset, and correction for blinks and eye movements was performed using a regression method (36). Artifact rejection was performed using a semi-automated procedure. Artifacts were defined as a voltage step of more than 50 μV, a difference of 300 μV within a trial, or a maximum difference of <0.5 μV within a 100-ms window; additional artifacts were identified manually. Segments were averaged separately for each image category and baseline corrected relative to the 200-ms pre-stimulus interval. The LPP was scored as the average activity from 400 to 1,000 ms at electrode site Pz (where the response was maximal), similar to previous reports (37–39). A difference score was calculated by subtracting neutral LPP from each emotional stimulus category (i.e., happy, sad, afraid, angry), and these difference scores were used in all analyses. Finally, the Average LPP was created by averaging LPP difference scores across all four emotion categories.

### Data analysis

Relationships between symptom severity (SAPS Reality Distortion, SAPS Disorganization, SANS Avolition, SANS Inexpressivity, and SCID depression) at each assessment point (concurrent and follow-up) and LPP difference scores were examined across the entire sample using separate regression models. Specifically, individual LPP difference scores were entered as the dependent variable and symptoms, group (coded: 0.5 for schizophrenia and −0.5 for affective psychosis), and their interaction were entered as independent variables. Altogether, the main analysis consisted of ten separate regressions, each including either a cross-sectional or follow-up symptom variable. Regression models aimed to quantify the association between LPP and symptom variables and not the prediction of LPP by symptoms, *per se*. A false discovery rate (FDR) correction was implemented to control for multiple comparisons within symptom clusters (40). Specifically, four FDR clusters were used (1) Cross-sectional mood including SANS Inexpressivity, SANS Avolition, and SCID Depression; (2) Cross sectional psychosis: SAPS Reality Distortion and SAPS Disorganization; (3) Follow-up mood: SANS Inexpressivity, SANS Avolition, and SCID Depression; (4) Follow-up psychosis: SAPS Reality Distortion and SAPS Disorganization.

## Results

### Demographics

The affective psychosis and schizophrenia spectrum groups were similar in regards to demographic variables (Table 1). Relative to the affective psychosis group, individuals in the schizophrenia spectrum group were more likely to be prescribed antipsychotic medications and showed more severe positive and negative symptoms, although not more severe depression. Significantly more variance was found in cross-sectional and lagged negative and positive symptoms but not depressive symptoms (see Supplemental materials for measures of variance; Table S9).

**Table 1:** Demographics.

	**SZ (*N* = 37)**	**AP (*N* = 37)**	**Statistic**	***p*-value**
Age	44.9 ± 7.8	44.3 ± 9.3	*t* = 0.3	0.76
Gender (% Male)	56.80%	59.50%	χ2 = 0.1	0.81
Ethnicity			χ2 = 1.4	0.7
% White	13.50%	5.40%		
% Black	8.10%	8.10%		
% Hispanic	75.70%	83.80%		
% Asian	2.70%	2.70%		
Medication Status			χ2 = 18.8	<0.001
% Antipsychotic	75.70%	27.00%		
**Symptom Severity**				
**(Cross-Sectional)**				
SAPS				
Disorganization Subscale	3.3 ± 5.3	1.7 ± 3.1	*t* = 1.7	0.11
Reality Distorting Subscale	3.2 ± 6.0	1.1 ± 3.9	*t =* 1.8	0.08
SANS				
Avolition Subscale	13.7 ± 8.1	4.81 ± 6.4	*t* = 5.2	<0.001
Inexpressivity Subscale	5.2 ± 7.1	1.78 ± 3.8	*t* = 2.6	0.01
SCID Depression Sum	11.1 ± 2.7	12.0 ± 3.9	*t* = −1.1	0.27
**Symptom Severity**				
**(Follow-up - 4 years later)**				
SAPS				
Disorganization Subscale	6.0 ± 7.5	3.0 ± 4.6	*t* = 2.0	0.048
Reality Distorting Subscale	8.6 ± 12.9	1.1 ± 2.4	*t* = 3.4	0.002
SANS				
Avolition Subscale	15.8 ± 9.4	8.4 ± 7.0	*t* = 3.7	<0.001
Inexpressivity Subscale	10.3 ± 9.7	2.7 ± 4.5	*t* = 4.1	<0.001
SCID Depression Total	11.5 ± 3.8	12.5 ± 4.5	*t* = −1.0	0.34
WRAT-3	45.3 ± 6.9	47.2 ± 5.3	*t* = −1.3	0.21

*SANS, Scale for the Assessment of Negative Symptoms; SAPS, Scale for the Assessment of Positive Symptoms; SCID Depression, Sum of SCID Depression Criterion A Scores. For these scales higher scores indicate greater impairment*.

### Group differences

**Figure 1:**
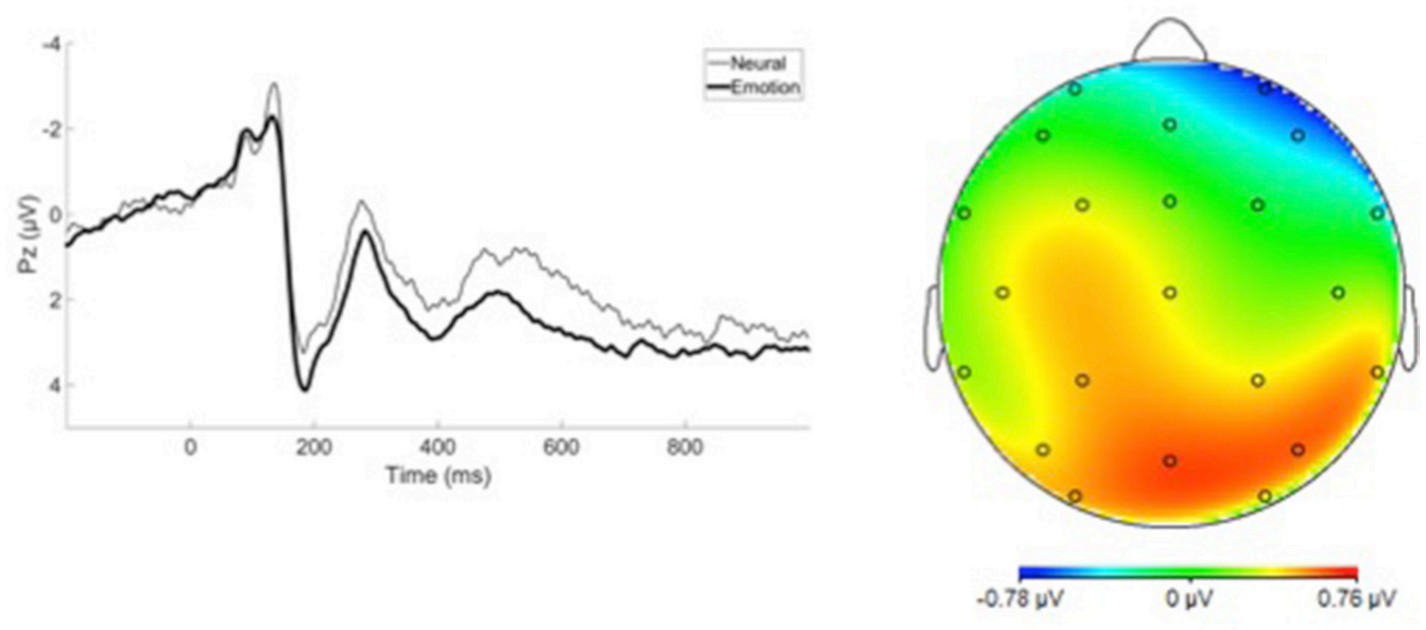
**(Left)** LPP Grand Average Waveform, **(Right)** Scalp Map illustrating LPP activation.

Grand average ERP waveforms are presented in Figure 1. No group differences were observed between affective psychosis and schizophrenia spectrum groups in LPP difference scores (Table 2). Antipsychotic medication also did not have a significant relationship to LPP (Table S1). In light of the similar mean-level LPP amplitude across groups, subsequent analyses focused on the Average LPP variable as a broader index of emotional reactivity (see the Supplemental Materials Table S2 for analyses conducted within emotion category).

**Table 2:** Group differences in LPP amplitude.

**LPP Difference Score Magnitude (**μ**V)**			
	**Schizophrenia mean (SD)**	**Affective psychosis mean (SD)**	***p*****-value**
Angry	0.57 (4.1)	0.65 (4.4)	0.93
Afraid	1.44 (3.17)	1.21 (5.6)	0.84
Sad	0.44 (5.0)	0.67 (4.5)	0.84
Happy	0.64 (4.2)	0.45(3.8)	0.84
Average	0.77 (3.0)	0.74 (3.9)	0.97

*SD, standard deviation*.

### Cross-sectional associations with symptoms

**Figure 2:**
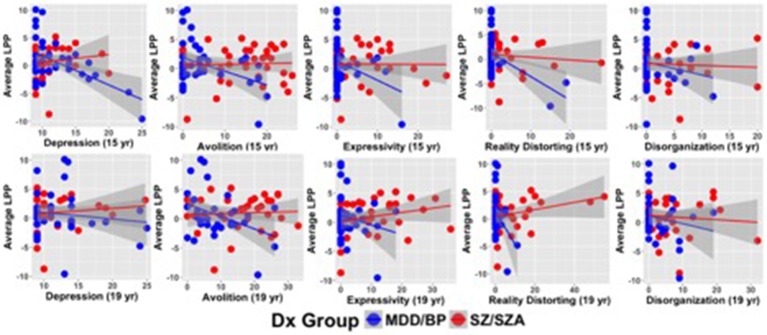
Scatterplots illustrating relationships between symptom severity and LPP difference scores averaged across emotion categories.

Table 3A describes the main effects of symptoms, group, and their interaction for each regression model (see Figure 2 for scatterplots; see Figure S1 for scatterplots illustrating relationship for SZ and SZA separately). Across these regression models, significant group effects were not observed. A significant symptom effect for psychotic symptoms (SAPS Reality Distortion) was observed such that greater symptom severity was associated with lower LPP amplitude, but all other symptom effects were non-significant. Broadly, associations between LPP difference score amplitude and symptom severity were more robust in the affective psychosis group compared to the schizophrenia group. Specifically, significant interactions between symptom variables and diagnostic group (coded: 0.5 for schizophrenia and −0.5 for affective psychosis) were observed for depressive (SCID Depression), negative (SANS Avolition), and psychotic (SAPS reality distortion) symptoms suggesting more robust negative associations between LPP and symptoms in the affective psychosis compared to the schizophrenia group. All significant interactions maintained significance when controlling for multiple comparisons (see Table S3 of Supplemental Materials for FDR-corrected *q*-values). The interaction between expressive negative symptoms (SANS Inexpressivity) and group was trend-level, and the interaction between disorganized symptoms (SAPS disorganization) and LPP was not significant (Table 3A). Regression models met the assumptions of normality of the residuals (See Tables S4, S5 of Supplemental Materials for skewness, kurtosis, and Shaprio-Wilk tests of the residuals for each model).

**Table 3a:** Multiple Regressions illustrating the cross-sectional association between Average LPP Difference Scores and symptoms, Group, and the interaction of group and symptoms.

**A.) Depression Regression**	**Beta**	**SE**	***t*-value**	***p*-value**
Group	0.139	0.763	0.182	0.856
SCID Depression	−0.194	0.121	−1.599	0.114
Group x Depression	−0.661	0.242	−2.732	0.008
**B.) Avolition Regression**	**Beta**	**SE**	***t*****-value**	***p*****-value**
Group	−0.996	0.94	−1.06	0.293
SANS Avolition	−0.11	0.057	−1.942	0.056
Group x Avolition	−0.251	0.113	−2.218	0.03
**C.) Inexpressivity Regression**	**Beta**	**SE**	***t*****-value**	***p*****-value**
Group	−0.6	0.856	−0.701	0.486
SANS Inexpressivity	−0.168	0.085	−1.973	0.053
Group x Inexpressivity	−0.331	0.171	−1.941	0.056
**D.) Disorganization Regression**	**Beta**	**SE**	***t*****-value**	***p*****-value**
Group	−0.247	0.845	−0.292	0.771
SAPS Disorganization	−0.129	0.11	−1.178	0.243
Group x Disorganization	−0.189	0.219	−0.86	0.393
**E.) Reality Distortion Regression**	**Beta**	**SE**	***t*****-value**	***p*****-value**
Group	−0.583	0.788	−0.739	0.462
SAPS Reality Distortion	−0.267	0.084	−3.178	0.002
Group x Reality Distortion	−0.426	0.168	−2.537	0.013

*Group was coded: 0.5 for schizophrenia and -0.5 for affective psychosis*.

### Symptoms at 4-year follow-up

Table 3B describes the main effects of symptoms (measured 4 years following ERP acquisition), group, and their interaction for each regression model (see Figure 2 for scatterplots; see Figure S1 for scatterplots illustrating relationship for SZ and SZA separately). Across these regression models, no significant effects of group were observed. A significant symptom effect for psychotic symptoms (SAPS Reality Distortion) and a trend-level effect for SANS Avolition were observed such that greater symptom severity was associated with lower LPP amplitude. All other symptom effects were non-significant. Significant interactions between symptom variables and diagnostic group (coded: 0.5 for schizophrenia and −0.5 for affective psychosis) were observed for negative (SANS Avolition) and psychotic (SAPS reality distortion) symptoms suggesting more robust negative lagged associations between LPP and symptoms in the affective psychosis compared to the schizophrenia group. Associations between LPP Amplitude and depressive, disorganized, or inexpressive negative symptoms did not maintain significance at follow-up. When controlling for multiple comparisons, only the interaction between psychotic symptoms and group maintained significance (see Table S3 of the Supplemental Materials for FDR-corrected *q*-values). Regression models met the assumptions of normality of the residuals (See Tables S4, S5 of Supplemental Materials for skewness, kurtosis, and Shaprio-Wilk tests of the residuals for each model).

**Table 3b:** Multiple regressions describing the lagged associations between Average LPP Difference Scores and Symptoms, Group, and the interaction of group and symptoms.

**A.) Depression Regression**	**Beta**	**SE**	***t*-value**	***p*-value**
Group	−0.229	0.856	−0.268	0.789
SCID Depression	−0.014	0.105	−0.133	0.894
Group x Depression	−0.2	0.211	−0.949	0.346
**B.) Avolition Regression**	**Beta**	**SE**	***t*****-value**	***p*****-value**
Group	−0.953	0.899	−1.06	0.293
SANS Avolition	−0.098	0.052	−1.896	0.062
Group x Avolition	−0.228	0.104	−2.202	0.031
**C.) Inexpressiviity Regression**	**Beta**	**SE**	***t*****-value**	***p*****-value**
Group	−0.575	0.999	−0.576	0.567
SANS Inexpressivity	−0.046	0.073	−0.63	0.531
Group x Inexpressivity	−0.239	0.146	−1.631	0.108
**D.) Disorganization Regression**	**Beta**	**SE**	***t*****-value**	***p*****-value**
Group	−0.5	0.878	−0.569	0.571
SAPS Disorganization	−0.087	0.077	−1.121	0.266
Group x Disorganization	−0.101	0.155	−0.649	0.518
**E.) Reality Distortion Regression**	**Beta**	**SE**	***t*****-value**	***p*****-value**
Group	−2.231	1.218	−1.831	0.072
SAPS Reality Distortion	−0.268	0.123	−2.186	0.032
Group x Reality Distortion	−0.668	0.245	−2.723	0.008

*Group was coded: 0.5 for schizophrenia and -0.5 for affective psychosis*.

### Further analysis of LPP difference scores

In order to aid in the interpretation of LPP difference scores, we conducted supplementary analyses to determine whether neural response to emotional category or neutral category was driving the observed relationships between LPP difference scores and symptoms (Tables S6a,b of Supplemental Materials). Specifically, we conducted multiple regressions where either LPP to averaged emotion faces or to neutral faces was the dependent variable and symptoms, group, and their interaction were the independent variable. For positive and depressive symptoms, significant interactions between symptoms and group were largely driven by increased LPP amplitude to neutral stimuli. For negative symptoms, significant interactions between symptoms and group were driven by both increased LPP amplitude to neutral stimuli and decreased LPP amplitude to emotional stimuli (Table S6).

### Role of antipsychotic medications

In order to better understand the role of antipsychotic dose in the aforementioned effects, we conducted similar multiple regressions to those described above but entered chlorpromazine (CPZ) equivalent dose as an additional independent variable [calculated using (41) documenting a score of zero for individuals not currently prescribed antipsychotics]. Critically, CPZ dose was not significantly associated with LPP in any of these models (see Tables S7, S8 of Supplemental Materials). Further, all aforementioned significant symptom by group interactions remained significant when including CPZ into the statistical model (see Tables S7, S8 of Supplemental Materials).

## Discussion

The goal of the current study was to test the hypothesis of transdiagnostic relationships between LPP amplitude and symptoms of emotional dysfunction, across putatively distinct psychotic illnesses. We found that significantly greater associations between LPP amplitude and symptom severity in individuals with affective psychosis compared to schizophrenia spectrum disorders for negative, depressive, and psychotic symptoms, thereby suggesting diagnostic specificity for these LPP-symptom relationships. Further, LPP amplitude showed some evidence of specificity to specific symptoms as no significant associations were observed between LPP amplitude and symptoms of disorganization.

Associations between reduced LPP amplitude and symptom severity in the affective psychosis group are consistent with several previous reports in non-psychotic depressed samples (12, 15) or in those at risk for developing depression (14, 16, 42). The current report extends prior findings by demonstrating LPP-to-symptom associations in those with psychotic disorders with a primary affective component suggesting that previous findings may generalize to both psychotic and non-psychotic forms of the illness, and further shows that these relationships for some symptom variables (i.e., avolition) persist over years. However, the current report also suggests that the LPP may be indexing more state-like compared to trait-like variability for depressive symptoms. Future studies of the LPP in major depressive disorder may benefit from more directly examining individuals with and without psychosis in the same sample, and further may benefit isolating avolition as a particularly relevant symptom dimension separate from global depression severity.

While LPP in individuals with affective psychosis was related to symptom severity measures, LPP was not related to any symptom measures in the schizophrenia group. This is consistent with previous studies who find similar LPP amplitude between healthy controls and those with schizophrenia, as well as studies that fail to find significant relationships between symptom severity and LPP in schizophrenia (13, 17). This raises interesting questions as to why LPP amplitude in those with schizophrenia appears insensitive to variations in clinical symptom measures. One possible explanation may be that the psychological and neural mechanisms that give rise to affective disturbance in schizophrenia may differ from affective psychosis. For example, in the moment emotional response may be intact in schizophrenia but decoupled from the retrospective reports of emotional experience measured via clinical interview, potentially due to difficulties encoding and retrieving affective experience (43, 44). In contrast, retrospective reports of emotional experience measured via clinical interview may be better coupled with in-the-moment emotional response in affective psychosis, potentially due to more intact cognitive ability in these individuals when compared to those with schizophrenia (45, 46).

Supplementary analyses revealed that associations between LPP and psychotic and depressive symptoms were largely driven by a larger LPP to neutral stimuli, a pattern that was also evident in the SZ group. In contrast, associations between LPP difference scores and negative symptoms were characterized by blunting of the discrimination between neutral and emotional faces. The associations between psychotic symptoms and neutral LPP in the current report are largely consistent with previous literature, including neuroimaging studies, which report enhanced activation of the limbic system to neutral faces in those at-risk for developing psychosis (47). Kapur and others have proposed that psychosis is associated with aberrant attributions of motivational salience to neutral stimuli (48). The current associations between neutral LPP and psychotic symptoms are consistent with this account. Further, there is some evidence that blunted amygdala responsivity to emotional faces in individuals with major depressive disorder is driven by increased reactivity to neutral faces and that this increased reactivity normalizes with treatment (49). The current results showing that LPP to neutral faces correlates with depressive symptoms are consistent with this account.”

### Limitations

The absence of a healthy comparison group limited our ability to interpret group differences between psychiatric groups and healthy controls. However, the aim of the current manuscript was to examine associations between the LPP and symptoms concurrently and years later across psychotic disorders. Further, previous studies have the LPP largely intact in schizophrenia making a healthy control group potentially less informative. Thus, a healthy comparison group was not integral to the examination of the aims of the current study. Second, although we found robust associations between symptoms, in our affective psychosis group, and LPP amplitude, we did not have the statistical power to examine such relationships within individual diagnoses (i.e., major depressive disorder, bipolar disorder). It may be the case that such relationships demonstrate important diagnostic group differences within affective psychosis and this remains an important question for future work. Further, may be important to separately analyze those with schizoaffective disorder. Finally, many of our participants were taking medications that have known influences on emotional processes, and the number of individuals prescribed such medications differed between groups. While medication status did not significantly modulate the relationship between symptoms and LPP amplitude, it remains possible that absence of effects in schizophrenia group may be due to the history of exposure to antipsychotics rather than being characteristic of the illness itself.

### Summary

We found modest evidence for negative associations between depressive and psychotic symptom severity and LPP amplitude. However, individuals with primary affective psychosis largely drove these correlations. Specifically, we found robust concurrent and lagged correlations of LPP with symptoms of psychotic, depressive, and negative symptoms not among individuals with schizophrenia but instead among individuals with a primary affective disorder with psychosis, providing some evidence for diagnostic specificity in the neural correlates of emotion dysfunction. These data are consistent with previous findings of robust correlations between depressive symptoms and reduced LPP amplitude in major depression (12), and also consistent with previous reports showing an absence of symptom-to-LPP relationships in those with schizophrenia (13, 17). Our results point to the possibility that similarly assessed symptom domains may arise from different psychological and neural mechanisms across psychotic disorders, underscoring the importance in studying multiple disorders together to further understand psychosis.

## Author contributions

RK implemented the data collection. AC wrote the initial version of the manuscript and conducted initial analyses. All authors provided comments on analyses and approved a final version of the manuscript.

### Conflict of interest statement

The authors declare that the research was conducted in the absence of any commercial or financial relationships that could be construed as a potential conflict of interest.

## Footnotes

^1^Development of the MacBrain Face Stimulus Set was overseen by Nim Tottenham and supported by the John D. and Catherine T. MacArthur Foundation Research Network on Early Experience and Brain Development. Please contact Nim Tottenham at tott0006@tc.umn.edu for more information concerning the stimulus set.

^2^The NimStim actors used were: 1, 2, 3, 5, 7, 8, 9, 10, 11, 12, 14, 17, 18, 20, 22, 23, 24, 25, 26, 27, 30, 34, 37, 38, 39, and 40.
