# Supplementary material for: Electrocortical Responses to Emotional Stimuli in Psychotic Disorders: Comparing Schizophrenia Spectrum Disorders and Affective Psychosis
Source: Front Psychiatry. 2018 Nov 16;9:586. doi: 10.3389/fpsyt.2018.00586 (PMC6250820; doi:10.3389/fpsyt.2018.00586)

S1:

**Antipsychotics predicting LPP**

|  | **Standardized Beta Values for Antipsychotics predicting LPP** | |
| --- | --- | --- |
|  | Schizophrenia Beta | Primary Affective Psychosis Beta |
| Angry | 0.29 | -0.14 |
| Afraid | -0.1 | -0.17 |
| Sad | 0.27 | -0.32 |
| Happy | 0.23 | -0.28 |
| Average | 0.26 | -0.26 |

All Beta Values were found to be not significant at a significance threshold of p < 0.05

**S2:**

**Raw Score Correlations**

|  |  | LPP Raw Score Values | | | | |
| --- | --- | --- | --- | --- | --- | --- |
|  |  | Happy | Sad | Afraid | Angry | Neutral |
| **Schizophrenia** |  |  |  |  |  |  |
| *Concurrent* | |  |  |  |  |  |
| SCID Depression | | 0.05 | 0.02 | -0.10 | -0.08 | -0.13 |
| SAPS Psychosis | | 0.15 | 0.37* | 0.32 | 0.11 | 0.34* |
| SAPS Disorganization | | 0.18 | 0.23 | 0.19 | 0.20 | 0.26 |
| SANS Inexpressivity | | 0.08 | -0.03 | -0.06 | 0.22 | 0.06 |
| SANS Avolition | | -0.09 | 0.27 | 0.06 | 0.18 | 0.09 |
| *Follow-Up (4-years later)* | | |  |  |  |  |
| SCID Depression | | 0.00 | 0.28 | 0.23 | 0.16 | 0.11 |
| SAPS Psychosis | | 0.21 | 0.23 | 0.15 | 0.20 | 0.00 |
| SAPS Disorganization | | 0.14 | 0.10 | 0.07 | 0.12 | 0.18 |
| SANS Inexpressivity | | 0.22 | 0.25 | -0.02 | 0.36* | 0.03 |
| SANS Avolition | | -0.23 | 0.23 | -0.09 | 0.21 | -0.01 |
| **Primary Affective Psychosis** | | |  |  |  |  |
| *Concurrent* | |  |  |  |  |  |
| SCID Depression | | 0.16 | 0.12 | -0.12 | 0.09 | 0.46** |
| SAPS Psychosis | | 0.24 | 0.18 | -0.09 | -0.09 | 0.43** |
| SAPS Disorganization | | 0.30 | 0.18 | 0.02 | -0.01 | 0.26 |
| SANS Inexpressivity | | -0.33* | -0.15 | -0.34* | -0.12 | 0.02 |
| SANS Avolition | | 0.00 | 0.07 | -0.22 | -0.03 | 0.25 |
| *Follow-Up (4-years later)* | | |  |  |  |  |
| SCID Depression | | 0.41* | 0.12 | 0.14 | -0.02 | 0.26 |
| SAPS Psychosis | | 0.32 | 0.18 | 0.04 | -0.05 | .040* |
| SAPS Disorganization | | 0.11 | -0.07 | -0.10 | -0.13 | 0.07 |
| SANS Inexpressivity | | -0.36* | -0.39* | -0.45** | -0.34* | -0.24 |
| SANS Avolition |  | 0.02 | -0.03 | -0.09 | -0.21 | 0.22 |

* p < 0.05; ** p < 0.01; SANS = Scale for the Assessment of Negative Symptoms; SAPS = Scale for the Assessment of Positive Symptoms; SCID Depression = Sum of SCID Depression Criterion A Scores.

**Table S3: FDR Corrected q-values for multiple regressions describing associations between of symptoms, group, and their interaction and Average LPP Difference Score.**

| FDR Correction |  |  |  |
| --- | --- | --- | --- |
|  | **Group** | **Symptom** | **Interaction** |
| **Cross-Sectional Mood Symptoms** | |  |  |
| SCID Depression | 0.856 | 0.114 | 0.024 |
| SANS Avolition | 0.729 | 0.084 | 0.045 |
| SANS Inexpressivity | 0.729 | 0.084 | 0.056 |
| **Cross-Sectional Psychotic Symptoms** | |  |  |
| SAPS Disorganization | 0.771 | 0.243 | 0.393 |
| SAPS Reality Distortion | 0.771 | 0.004 | 0.026 |
|  |  |  |  |
| **Lagged Mood Symptoms** |  |  |  |
| SCID Depression | 0.789 | 0.894 | 0.346 |
| SANS Avolition | 0.789 | 0.186 | 0.093 |
| SANS Inexpressivity | 0.789 | 0.7965 | 0.162 |
| **Lagged Psychotic Symptoms** |  |  |  |
| SAPS Disorganization | 0.571 | 0.266 | 0.518 |
| SAPS Reality Distortion | 0.144 | 0.064 | 0.016 |

**Note: group was coded: 0.5 for schizophrenia and -0.5 for affective psychosis**

**Table S4: Skewness and Kurtosis of the residual for each regression model**

| **Regression Residual** | **Skewness** | **Kurtosis** |
| --- | --- | --- |
|  | **Statistic** | **Statistic** |
| ***Concurrent*** |  |  |
| **SCID Depression** | **-0.11** | **0.573** |
| **SAPS Reality Distortion** | **0.184** | **0.961** |
| **SAPS Disorganization** | **-0.128** | **1.423** |
| **SANS Inexpressivity** | **0.036** | **0.475** |
| **SANS Avolition** | **-0.079** | **0.415** |
| ***Follow-Up (4-years later)*** |  |  |
| **SCID Depression** | **-0.033** | **1.757** |
| **SAPS Reality Distortion** | **0.131** | **1.1** |
| **SAPS Disorganization** | **0.042** | **1.438** |
| **SANS Inexpressivity** | **-0.005** | **0.902** |
| **SANS Avolition** | **-0.088** | **0.942** |

**Table S5: Shaprio-Wilk Tests of the residual for each regression model**

| **Regression Residual** | **Shapiro-Wilk** |
| --- | --- |
| ***Concurrent*** | **p-value** |
| **SCID Depression** | **0.735** |
| **SAPS Reality Distortion** | **0.39** |
| **SAPS Disorganization** | **0.108** |
| **SANS Inexpressivity** | **0.783** |
| **SANS Avolition** | **0.863** |
| ***Follow-Up (4-years later)*** |  |
| **SCID Depression** | **0.05** |
| **SAPS Reality Distortion** | **0.419** |
| **SAPS Disorganization** | **0.102** |
| **SANS Inexpressivity** | **0.383** |
| **SANS Avolition** | **0.513** |

Table S6a: Multiple regression analyses describing the cross-sectional associations between LPP to Averaged Emotion faces (left) and LPP to Neutral faces (right) and group, symptoms, and their interaction.

| A.) Depression Regression | **LPP to Averaged Emotion Faces** | | | | | | |  | **LPP to Neutral Faces** | | | | | | | |  |
| --- | --- | --- | --- | --- | --- | --- | --- | --- | --- | --- | --- | --- | --- | --- | --- | --- | --- |
|  | **beta** | **SE** | | **t-value** | | **p-value** | |  | **beta** | | **SE** | | **t-value** | | **p-value** | |  |
| **Group** | 1.757 | 0.923 | | 1.903 | | 0.061 | |  | 1.618 | | 1.034 | | 1.565 | | 0.122 | |  |
| **SCID Depression** | 0.006 | 0.146 | | 0.042 | | 0.967 | |  | 0.2 | | 0.164 | | 1.218 | | 0.227 | |  |
| **Group x Depression** | 0.127 | 0.293 | | 0.432 | | 0.667 | |  | 0.788 | | 0.328 | | 2.403 | | 0.019 | |  |
|  |  |  | |  | |  | |  |  | |  | |  | |  | |  |
| B.) Avolition Regression | **LPP to Averaged Emotion Faces** | | | | | | |  | **LPP to Neutral Faces** | | | | | | | |  |
|  | **beta** | **SE** | | **t-value** | | **p-value** | |  | **beta** | | **SE** | | **t-value** | | **p-value** | |  |
| **Group** | 1.861 | | 1.078 | | 1.727 | | 0.089 | |  | 2.857 | | 1.276 | | 2.24 | | 0.028 | |
| **SANS Avolition** | 0.01 | | 0.065 | | 0.162 | | 0.872 | |  | 0.12 | | 0.077 | | 1.567 | | 0.122 | |
| **Group x Avolition** | -0.099 | | 0.13 | | -0.765 | | 0.447 | |  | 0.152 | | 0.153 | | 0.987 | | 0.327 | |
|  |  |  | |  | |  | |  |  | |  | |  | |  | |  |
| C.) Inexpressivity Regression | **LPP to Averaged Emotion Faces** | | | | | | |  | **LPP to Neutral Faces** | | | | | | | |  |
|  | **beta** | **SE** | | **t-value** | | **p-value** | |  | **beta** | | **SE** | | **t-value** | | **p-value** | |  |
| **Group** | 1.304 | 0.949 | | 1.374 | | 0.174 | |  | 1.903 | | 1.168 | | 1.629 | | 0.108 | |  |
| **SANS Inexpressivity** | -0.136 | 0.095 | | -1.441 | | 0.154 | |  | 0.032 | | 0.116 | | 0.275 | | 0.784 | |  |
| **Group x Inexpressivity** | -0.336 | 0.189 | | -1.776 | | 0.08 | |  | -0.005 | | 0.233 | | -0.021 | | 0.983 | |  |
|  |  |  | |  | |  | |  |  | |  | |  | |  | |  |
| D.) **Reality Distortion Regression** | **LPP to Averaged Emotion Faces** | | | | | | |  | **LPP to Neutral Faces** | | | | | | | |  |
|  | **beta** | **SE** | | **t-value** | | **p-value** | |  | **beta** | | **SE** | | **t-value** | | **p-value** | |  |
| **Group** | 2.029 | 0.919 | | 2.207 | | 0.031 | |  | 2.612 | | 1.037 | | 2.52 | | 0.014 | |  |
| **SAPS Reality Distortion** | 0.127 | 0.098 | | 1.299 | | 0.198 | |  | 0.394 | | 0.11 | | 3.569 | | 0.001 | |  |
| **Group x Reality Distortion** | -0.111 | 0.196 | | -0.568 | | 0.572 | |  | 0.315 | | 0.221 | | 1.426 | | 0.158 | |  |
|  |  |  | |  | |  | |  |  | |  | |  | |  | |  |
| E.) Disorganization Regression | **LPP to Averaged Emotion Faces** | | | | | | |  | **LPP to Neutral Faces** | | | | | | | |  |
|  | **beta** | **SE** | | **t-value** | | **p-value** | |  | **beta** | | **SE** | | **t-value** | | **p-value** | |  |
| **Group** | 2.064 | 0.92 | | 2.244 | | 0.028 | |  | 2.312 | | 1.089 | | 2.122 | | 0.037 | |  |
| **SAPS Disorganization** | 0.18 | 0.119 | | 1.506 | | 0.137 | |  | 0.309 | | 0.141 | | 2.185 | | 0.032 | |  |
| **Group x Disorganization** | 0.023 | 0.238 | | 0.098 | | 0.922 | |  | 0.212 | | 0.282 | | 0.751 | | 0.456 | |  |

**Note: group was coded: 0.5 for schizophrenia and -0.5 for affective psychosis**

**Table S6b: Multiple regression describing the lagged associations between LPP to Averaged Emotion faces (left) and LPP to Neutral faces (right) and group, symptoms, and their interaction.**

|  | **LPP to Averaged Emotion Faces** | | | | | | | |  | | **LPP to Neutral Faces** | | | | | | | | | | |  |  |  |  |
| --- | --- | --- | --- | --- | --- | --- | --- | --- | --- | --- | --- | --- | --- | --- | --- | --- | --- | --- | --- | --- | --- | --- | --- | --- | --- |
| A.) Depression Regression | **beta** | | **SE** | | **t-value** | | **p-value** | |  | **beta** | | **SE** | | **t-value** | | | | **p-value** | | | | |  |  |  |
| **Group** | 1.513 | | 0.929 | | 1.629 | | 0.108 | |  | 1.742 | | 1.114 | | | 1.564 | | | | 0.123 | | | | |  |  |
| **SCID Depression** | 0.192 | | 0.114 | | 1.676 | | 0.099 | |  | 0.206 | | 0.137 | | | 1.5 | | | | 0.138 | | | | |  |  |
| **Group x Depression** | -0.017 | | 0.229 | | -0.073 | | 0.942 | |  | 0.183 | | 0.274 | | | 0.668 | | | | 0.506 | | | | |  |  |
|  |  | |  | |  | |  | |  |  | |  | |  | | | |  | | | | |  |  |  |
| B.) Avolition Regression | **LPP to Averaged Emotion Faces** | | | | | | | |  | **LPP to Neutral Faces** | | | | | | | | | | | | |  |  |  |
|  | **beta** | | **SE** | | **t-value** | | **p-value** | |  | **beta** | | **SE** | | **t-value** | | | | **p-value** | | | | |  |  |  |
| **Group** | 1.555 | | 1.037 | | 1.5 | | 0.139 | |  | 2.508 | | 1.23 | | | | 2.038 | | | | 0.046 | | | | |  |
| **SANS Avolition** | -0.02 | | 0.06 | | -0.333 | | 0.74 | |  | 0.078 | | 0.071 | | | | 1.105 | | | | 0.273 | | | | |  |
| **Group x Avolition** | -0.065 | | 0.12 | | -0.544 | | 0.588 | |  | 0.163 | | 0.142 | | | | 1.151 | | | | 0.254 | | | | |  |
|  |  | |  | |  | |  | |  |  | |  | |  | | | |  | | | | |  |  |  |
| C.) inexpressivity Regression | **LPP to Averaged Emotion Faces** | | | | | | | |  | **LPP to Neutral Faces** | | | | | | | | | | | | |  |  |  |
|  | **beta** | | **SE** | | **t-value** | | **p-value** | |  | **beta** | | **SE** | | **t-value** | | | | **p-value** | | | | |  |  |  |
| **Group** | 0.403 | | 1.033 | | 0.39 | | 0.698 | |  | 0.978 | | | 1.327 | | | | 0.737 | | | | 0.464 | | | | |
| **SANS Inexpressivity** | -0.176 | | 0.076 | | -2.327 | | 0.023 | |  | -0.13 | | | 0.097 | | | | -1.337 | | | | 0.186 | | | | |
| **Group x Inexpressivity** | -0.524 | | 0.151 | | -3.464 | | 0.001 | |  | -0.285 | | | 0.194 | | | | -1.468 | | | | 0.147 | | | | |
|  |  | |  | |  | |  | |  |  | |  | |  | | | |  | | | | |  |  |  |
| D.) Reality Distortion Regression | **LPP to Averaged Emotion Faces** | | | | | | | |  | **LPP to Neutral Faces** | | | | | | | | | | | | |  |  |  |
|  | **beta** | | **SE** | | **t-value** | | **p-value** | |  | **beta** | | **SE** | | **t-value** | | | | **p-value** | | | | |  |  |  |
| **Group** | 2.942 | | 1.398 | | 2.104 | | 0.039 | |  | 5.173 | | 1.623 | | | 3.187 | | | | 0.002 | | | | |  |  |
| **SAPS Reality Distortion** | 0.166 | | 0.141 | | 1.18 | | 0.242 | |  | 0.434 | | 0.163 | | | 2.657 | | | | 0.01 | | | | |  |  |
| **Group x Reality Distortion** | 0.202 | | 0.281 | | 0.718 | | 0.476 | |  | 0.869 | | 0.327 | | | 2.662 | | | | 0.01 | | | | |  |  |
|  |  | |  | |  | |  | |  |  | |  | |  | | | |  | | | | |  |  |  |
| E.) Disorganization Regression | **LPP to Averaged Emotion Faces** | | | | | | | |  | **LPP to Neutral Faces** | | | | | | | | | | | | |  |  |  |
|  | **beta** | | **SE** | | **t-value** | | **p-value** | |  | **beta** | | **SE** | | **t-value** | | | | **p-value** | | | | |  |  |  |
| **Group** | 1.703 | | 0.972 | | 1.753 | | 0.084 | |  | 2.203 | | 1.164 | | | | 1.892 | | | | 0.063 | | | | |  |
| **SAPS Disorganization** | 0.001 | | 0.086 | | 0.008 | | 0.994 | |  | 0.087 | | 0.103 | | | | 0.853 | | | | 0.397 | | | | |  |
| **Group x Disorganization** | -0.121 | 0.171 | | -0.704 | | 0.484 | |  | | -0.02 | | 0.205 | | | | -0.098 | | | | 0.922 | | | | |  |

**Note: group was coded: 0.5 for schizophrenia and -0.5 for affective psychosis**

**Table S7: Pearson Correlations between CPZ Equivalents and LPP Difference Scores**

|  | **CPZ Dosage with Antipsychotic Absent Participants** |
| --- | --- |
| **Average LPP Difference Score** | -0.085 |

**Table S8a: Multiple Regressions describing the cross-sectional association between Average LPP Difference Scores and Cross-sectional Symptoms, Group, CPZ Dose and the interaction of group and symptoms.**

| **A.) Depression Regression** | **beta** | **SE** | **t-value** | **p-value** |
| --- | --- | --- | --- | --- |
| **Group** | 0.399 | 0.881 | 0.453 | 0.652 |
| **CPZ Dose** | 0 | 0.001 | 0.213 | 0.832 |
| **SCID Depression** | -0.188 | 0.131 | -1.432 | 0.157 |
| **Group x Depression** | -0.72 | 0.258 | -2.785 | 0.007 |
|  |  |  |  |  |
| **B.) Reality Distortion Regression** | **beta** | **SE** | **t-value** | **p-value** |
| **Group** | -0.411 | 0.885 | -0.464 | 0.644 |
| **CPZ Dose** | 0 | 0.001 | 0.222 | 0.825 |
| **SAPS Reality Distortion** | -0.295 | 0.09 | -3.261 | 0.002 |
| **Group x Reality Distortion** | -0.389 | 0.185 | -2.1 | 0.04 |
|  |  |  |  |  |
| **C.) Disorganization Regression** | **beta** | **SE** | **t-value** | **p-value** |
| **Group** | -0.326 | 0.966 | -0.337 | 0.737 |
| **CPZ Dose** | -0.001 | 0.001 | -0.685 | 0.496 |
| **SAPS Disorganization** | -0.145 | 0.118 | -1.231 | 0.223 |
| **Group x Disorganization** | -0.167 | 0.236 | -0.708 | 0.481 |
|  |  |  |  |  |
| **D.) Avolition Regression** | **beta** | **SE** | **t-value** | **p-value** |
| **Group** | -0.863 | 1.013 | -0.852 | 0.398 |
| **CPZ Dose** | 1.74E-05 | 0.001 | 0.015 | 0.988 |
| **SANS Avolition** | -0.113 | 0.062 | -1.832 | 0.072 |
| **Group x Avolition** | -0.244 | 0.123 | -1.986 | 0.051 |
|  |  |  |  |  |
| **E.) Inexpressivity Regression** | **beta** | **SE** | **t-value** | **p-value** |
| **Group** | -0.518 | 0.948 | -0.547 | 0.587 |
| **CPZ Dose** | 0.001 | 0.001 | 0.475 | 0.636 |
| **SANS Inexpressivity** | -0.189 | 0.114 | -1.663 | 0.101 |
| **Group x Inexpressivity** | -0.49 | 0.227 | -2.161 | 0.035 |

**Note: group was coded: 0.5 for schizophrenia and -0.5 for affective psychosis**

**Table S8b: Multiple Regressions describing the lagged associations betweeen Average LPP Difference Scores and Symptoms, Group, CPZ Dose and the interaction of group and symptoms**

| **A.) Depression Regression** | **beta** | **SE** | **t-value** | **p-value** |
| --- | --- | --- | --- | --- |
| **Group** | -0.413 | 1.007 | -0.41 | 0.683 |
| **CPZ Dose** | -0.001 | 0.001 | -0.845 | 0.402 |
| **SCID Depression** | 0.007 | 0.118 | 0.055 | 0.956 |
| **Group x Depression** | -0.271 | 0.236 | -1.146 | 0.257 |
|  |  |  |  |  |
| **B.) Reality Distortion Regression** | **beta** | **SE** | **t-value** | **p-value** |
| **Group** | -2.209 | 1.312 | -1.684 | 0.098 |
| **CPZ Dose** | 0 | 0.001 | -0.342 | 0.733 |
| **SAPS Reality Distortion** | -0.266 | 0.134 | -1.983 | 0.052 |
| **Group x Reality Distortion** | -0.648 | 0.266 | -2.441 | 0.018 |
|  |  |  |  |  |
| **C.) Disorganization Regression** | **beta** | **SE** | **t-value** | **p-value** |
| **Group** | -0.512 | 1.015 | -0.504 | 0.616 |
| **CPZ Dose** | -0.001 | 0.001 | -0.501 | 0.618 |
| **SAPS Disorganization** | -0.079 | 0.085 | -0.931 | 0.356 |
| **Group x Disorganization** | -0.107 | 0.164 | -0.653 | 0.516 |
|  |  |  |  |  |
| **D.) Avolition Regression** | **beta** | **SE** | **t-value** | **p-value** |
| **Group** | -0.797 | 0.998 | -0.799 | 0.428 |
| **CPZ Dose** | 0 | 0.001 | 0.098 | 0.923 |
| **SANS Avolition** | -0.103 | 0.06 | -1.703 | 0.094 |
| **Group x Avolition** | -0.237 | 0.115 | -2.067 | 0.043 |
|  |  |  |  |  |
| **E.) Inexpressivity Regression** | **beta** | **SE** | **t-value** | **p-value** |
| **Group** | -0.318 | 1.095 | -0.291 | 0.772 |
| **CPZ Dose** | 0 | 0.001 | -0.329 | 0.744 |
| **SANS Inexpressivity** | -0.009 | 0.084 | -0.102 | 0.919 |
| **Group x Inexpressivity** | -0.27 | 0.167 | -1.615 | 0.112 |

**Note: group was coded: 0.5 for schizophrenia and -0.5 for affective psychosis**

**Table S9: Variability in predictor variables for Affective Psychosis and Schizophrenia Groups**

|  | **Schizophrenia** | | **Affective Psychosis** | | **Levene's Test for Equality of Variance** | | |
| --- | --- | --- | --- | --- | --- | --- | --- |
|  | **Variance** | **Range** | **Variance** | **Range** | **f-value** | **p-value** | |
| ***Cross-Sectional*** |  |  |  |  |  |  |  |
| SCID Depression | 7.45 | 11 | 15.471 | 16 | 3.49 | 0.066 |  |
| SAPS Reality Distortion | 34.886 | 28 | 15.465 | 19 | 5.28 | 0.025 |  |
| SAPS Disorganization | 28.229 | 20 | 9.734 | 12 | 5.808 | 0.019 |  |
| SANS Inexpressivity | 49.761 | 27 | 14.73 | 16 | 13.572 | <0.001 |  |
| SANS Avolition | 66.263 | 26 | 40.88 | 20 | 4.166 | 0.045 |  |
| ***Lagged Symptoms*** |  |  |  |  |  |  |  |
| SCID Depression | 14.555 | 15.75 | 19.867 | 16 | 0.51 | 0.477 |  |
| SAPS Reality Distortion | 167.569 | 55 | 5.669 | 11 | 21.815 | <0.001 |  |
| SAPS Disorganization | 56.151 | 32 | 21.087 | 19 | 3.143 | 0.081 |  |
| SANS Inexpressivity | 94.564 | 36 | 20.255 | 18 | 16.734 | <0.001 |  |
| SANS Avolition | 87.865 | 33 | 49.546 | 26 | 4.687 | 0.034 |  |

**Figure 1: Scatterplots for SZA and SZ subjects shown separately**


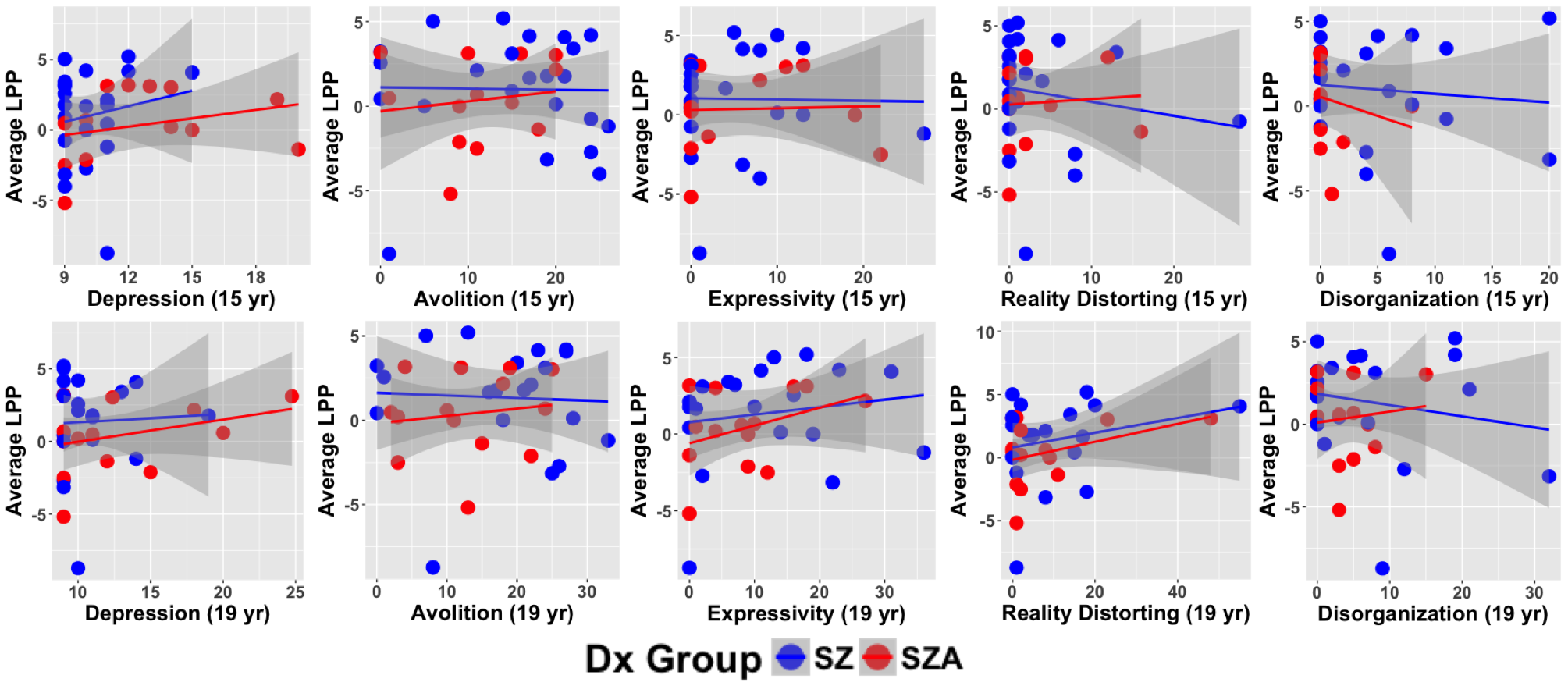

Supplement: Supplementary file 1 [file Data_Sheet_1.docx]
